# LNCaP Atlas: Gene expression associated with *in vivo *progression to castration-recurrent prostate cancer

**DOI:** 10.1186/1755-8794-3-43

**Published:** 2010-09-24

**Authors:** Tammy L Romanuik, Gang Wang, Olena Morozova, Allen Delaney, Marco A Marra, Marianne D Sadar

**Affiliations:** 1 Genome Sciences Centre, British Columbia Cancer Agency, Vancouver, British Columbia, Canada

## Abstract

**Background:**

There is no cure for castration-recurrent prostate cancer (CRPC) and the mechanisms underlying this stage of the disease are unknown.

**Methods:**

We analyzed the transcriptome of human LNCaP prostate cancer cells as they progress to CRPC *in vivo *using replicate LongSAGE libraries. We refer to these libraries as the LNCaP atlas and compared these gene expression profiles with current suggested models of CRPC.

**Results:**

Three million tags were sequenced using *in vivo *samples at various stages of hormonal progression to reveal 96 novel genes differentially expressed in CRPC. Thirty-one genes encode proteins that are either secreted or are located at the plasma membrane, 21 genes changed levels of expression in response to androgen, and 8 genes have enriched expression in the prostate. Expression of 26, 6, 12, and 15 genes have previously been linked to prostate cancer, Gleason grade, progression, and metastasis, respectively. Expression profiles of genes in CRPC support a role for the transcriptional activity of the androgen receptor (*CCNH, CUEDC2, FLNA, PSMA7*), steroid synthesis and metabolism (*DHCR24, DHRS7*, *ELOVL5, HSD17B4*, *OPRK1*), neuroendocrine (*ENO2, MAOA, OPRK1, S100A10, TRPM8*), and proliferation (*GAS5*, *GNB2L1*, *MT-ND3*, *NKX3-1*, *PCGEM1*, *PTGFR*, *STEAP1*, *TMEM30A*), but neither supported nor discounted a role for cell survival genes.

**Conclusions:**

The *in vivo *gene expression atlas for LNCaP was sequenced and support a role for the androgen receptor in CRPC.

## Background

Systemic androgen-deprivation therapy by orchiectomy or agonists of gonadotropic releasing hormone are routinely used to treat men with metastatic prostate cancer to reduce tumor burden and pain. This therapy is based on the dependency of prostate cells for androgens to grow and survive. The inability of androgen-deprivation therapy to completely and effectively eliminate all metastatic prostate cancer cell populations is manifested by a predictable and inevitable relapse, referred to as castration-recurrent prostate cancer (CRPC). CRPC is the end stage of the disease and fatal to the patient within 16-18 months of onset.

The mechanisms underlying progression to CRPC are unknown. However, there are several models to explain its development. One such model indicates the involvement of the androgen signaling pathway[1-4]. Key to this pathway is the androgen receptor (AR) which is a steroid hormone receptor and transcription factor. Mechanisms of progression to CRPC that involve or utilize the androgen signaling pathway include: hypersensitivity due to *AR *gene amplification [5,6]; changes in AR co-regulators such as nuclear receptor coactivators (*NCOA1 *and *NCOA2*) [7,8]; intraprostatic *de novo *synthesis of androgen[9] or metabolism of AR ligands from residual adrenal androgens[10,11]; AR promiscuity of ligand specificity due to mutations[12]; and ligand-independent activation of AR by growth factors [protein kinase A (PKA), interleukin 6 (IL6), and epidermal growth factor (EGF)][13-15]. Activation of the AR can be determined by assaying for the expression of target genes such as prostate-specific antigen (PSA)[16]. Other models of CRPC include the neuroendocrine differentiation [17], the stem cell model [18] and the imbalance between cell growth and cell death [3]. It is conceivable that these models may not mutual exclusive. For example altered AR activity may impact cell survival and proliferation.

Here, we describe long serial analysis of gene expression (LongSAGE) libraries[19,20] made from RNA sampled from biological replicates of the *in vivo *LNCaP Hollow Fiber model of prostate cancer as it progresses to the castration-recurrent stage. Gene expression signatures that were consistent among the replicate libraries were applied to the current models of CRPC.

## Methods

### *In vivo *LNCaP Hollow Fiber model

The LNCaP Hollow Fiber model of prostate cancer was performed as described previously[21-23]. All animal experiments were performed according to a protocol approved by the Committee on Animal Care of the University of British Columbia. Serum PSA levels were determined by enzymatic immunoassay kit (Abbott Laboratories, Abbott Park, IL, USA). Fibers were removed on three separate occasions representing different stages of hormonal progression that were androgen-sensitive (AS), responsive to androgen-deprivation (RAD), and castration-recurrent (CR). Samples were retrieved immediately prior to castration (AS), as well as 10 (RAD) and 72 days (CR) post-surgical castration.

### RNA sample generation, processing, and quality control

Total RNA was isolated immediately from cells harvested from the *in vivo *Hollow Fiber model using TRIZOL Reagent (Invitrogen) following the manufacturer's instructions. Genomic DNA was removed from RNA samples with DNaseI (Invitrogen). RNA quality and quantity were assessed by the Agilent 2100 Bioanalyzer (Agilent Technologies, Mississauga, ON, Canada) and RNA 6000 Nano LabChip kit (Caliper Technologies, Hopkinton, MA, USA).

### Quantitative real-time polymerase chain reaction

Oligo-d(T)-primed total RNAs (0.5 μg per sample) were reverse-transcribed with SuperScript III (Invitrogen Life Technologies, Carlsbad, CA, USA). An appropriate dilution of cDNA and gene-specific primers were combined with SYBR Green Supermix (Invitrogen) and amplified in ABI 7900 real-time PCR machine (Applied Biosystems, Foster City, CA, USA). All qPCR reactions were performed in triplicate. The threshold cycle number (Ct) and expression values with standard deviations were calculated in Excel. Primer sequences for real-time PCRs are: *KLK3*, F': 5'-CCAAGTTCATGCTGTGTGCT-3' and R:' 5'-CCCATGACGTGATACCTTGA-3'; glyceraldehyde-3-phosphate (*GAPDH*), F': 5'-CTGACTTCAACAGCGACACC-3' and R:' 5'-TGCTGTAGCCAAATTCGTTG-3'). Real-time amplification was performed with initial denaturation at 95°C for 2 min, followed by 40 cycles of two-step amplification (95°C for 15 sec, 55°C for 30 sec).

### LongSAGE library production and sequencing

RNA from the hollow fibers of three mice (biological replicates) representing different stages of prostate cancer progression (AS, RAD, and CR) were used to make a total of nine LongSAGE libraries. LongSAGE libraries were constructed and sequenced at the Genome Sciences Centre, British Columbia Cancer Agency. Five micrograms of starting total RNA was used in conjunction with the Invitrogen I-SAGE Long kit and protocol with alterations [24]. Raw LongSAGE data are available at Gene Expression Omnibus [25] as series accession number GSE18402. Individual sample accession numbers are as follows: S1885, GSM458902; S1886, GSM458903; S1887, GSM458904; S1888, GSM458905; S1889, GSM458906; S1890, GSM458907; S1891, GSM458908; S1892, GSM458909; and S1893, GSM458910.

### Gene expression analysis

LongSAGE expression data was analyzed with DiscoverySpace 4.01 software [26]. Sequence data were filtered for bad tags (tags with one N-base call) and linker-derived tags (artifact tags). Only LongSAGE tags with a sequence quality factor (QF) greater than 95% were included in analysis. The phylogenetic tree was constructed with a distance metric of 1-r (where "r" equals the Pearson correlation coefficient). Correlations were computed (including tag counts of zero) using the Regress program of the Stat package written by Ron Perlman, and the tree was optimized using the Fitch program[27] in the Phylip package[28]. Graphics were produced from the tree files using the program TreeView[29]. Tag clustering analysis was performed using the Poisson distribution-based K-means clustering algorithm. The K-means algorithm clusters tags based on count into 'K' partitions, with the minimum intracluster variance. PoissonC was developed specifically for the analysis of SAGE data [30]. The java implementation of the algorithm was kindly provided by Dr. Li Cai (Rutgers University, NJ, USA). An optimal value for K (K = 10) was determined [31].

### Principle component analysis

Principle component analysis was performed using GeneSpring™ software version 7.2 (Silicon Genetics, CA). Affymetrix datasets of clinical prostate cancer and normal tissue were downloaded from Gene Expression Omnibus [25] (accession numbers: GDS1439 and GDS1390) and analyzed in GeneSpring™. Of the 96 novel CR-associated genes, 76 genes had corresponding Affymetrix probe sets. These probe sets were applied as the gene signature in this analysis. Principle component (PC) scores were calculated according to the standard correlation between each condition vector and each principle component vector.

## Results

### LongSAGE library and tag clustering

RNA isolated from the LNCaP Hollow Fiber model was obtained from at least three different mice (13N, 15N, and 13R; biological replicates) at three stages of cancer progression that were androgen-sensitive (AS), responsive to androgen-deprivation (RAD), and castration-recurrent (CR). To confirm that the samples represented unique disease-states, we determined the levels of *KLK3 *mRNA, a biomarker that correlates with progression, using quantitative real time-polymerase chain reaction (qRT-PCR). As expected, *KLK3 *mRNA levels dropped in the stage of cancer progression that was RAD versus AS (58%, 49%, and 37%), and rose in the stage of cancer progression that was CR versus RAD (229%, 349%, and 264%) for mice 13R, 15N, and 13N, respectively (Additional file 1). Therefore, we constructed nine LongSAGE libraries, one for each stage and replicate.

LongSAGE libraries were sequenced to 310,072 - 339,864 tags each, with a combined total of 2,931,124 tags, and filtered to leave only useful tags for analysis (Table 1). First, bad tags were removed because they contain at least one N-base call in the LongSAGE tag sequence. The sequencing of the LongSAGE libraries was base called using PHRED software. Tag sequence-quality factor (QF) and probability was calculated to ascertain which tags contain erroneous base-calls. The second line of filtering removed LongSAGE tags with probabilities less than 0.95 (QF < 95%). Linkers were introduced into SAGE libraries as known sequences utilized to amplify ditags prior to concatenation. At a low frequency, linkers ligate to themselves creating linker-derived tags (LDTs). These LDTs do not represent transcripts and were removed from the LongSAGE libraries. A total of 2,305,589 useful tags represented by 263,197 tag types remained after filtering. Data analysis was carried out on this filtered data.

**Table 1:** Composition of LongSAGE libraries

Library	S1885	S1886	S1887	S1888	S1889	S1890	S1891	S1892	S1893
Mouse-Condition	13N-AS*	13N-RAD†	13N-CR‡	15N-AS	15N-RAD	15N-CR	13R-AS	13R-RAD	13R-CR
Unfiltered Total Tags	310,516	318,102	339,864	338,210	310,072	326,870	337,546	314,440	335,504
No. of Bad Tags	955	1,010	1,083	1,097	983	737	900	744	832

Minus Bad Tags									

Total Tags	309,561	317,092	338,781	337,113	309,089	326,133	336,646	313,696	334,672
Tag Types	79,201	96,973	99,730	81,850	84,499	88,249	79,859	91,438	90,675
No. of Duplicate Ditags	19,761	12,220	12,678	21,973	17,471	12,836	24,552	12,786	13,127
% of Duplicate Ditags	6.38	3.85	3.74	6.52	5.65	3.94	7.29	4.08	3.92
Average QF§ of Tags	0.85	0.88	0.87	0.86	0.89	0.88	0.88	0.80	0.87
No. of Tags QF < 0.95	63,057	62,872	71,576	68,993	54,627	54,470	68,981	101,215	69,647

Q ≥ 0.95									

Total Tags	246,504	254,220	267,205	268,120	254,462	271,663	267,665	212,481	265,025
Tag Types	52,033	67,542	66,748	52,606	59,374	64,985	53,715	54,682	64,837
Total Tags Combined					2,307,345				
Tag Types Combined					263,199				
No. of LDTs II Type I	124	72	174	179	84	186	164	118	301
No. of LDTs Type II	19	9	54	56	33	40	60	24	59

Minus LDTs									

Total Tags	246,361	254,139	266,977	267,885	254,345	271,437	267,441	212,339	264,665
Tag Types	52,031	67,540	66,746	52,604	59,372	64,983	53,713	54,680	64,835
Total Tags Combined					2,305,589				
Tag Types Combined					263,197				

* AS, Androgen-sensitive

† RAD, Responsive to androgen-deprivation

‡ CR, Castration-recurrent

§ QF, Quality Factor

II LDTs, Linker-derived tags

**Figure 1:**
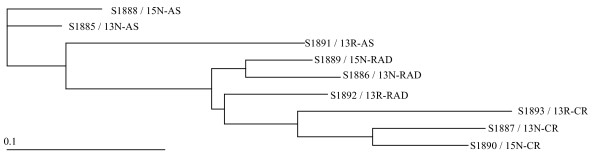
**Clustering of the nine LongSAGE libraries in a hierarchical tree**. The tree was generated using a Pearson correlation-based hierarchical clustering method and visualized with TreeView. LongSAGE libraries constructed from similar stages of prostate cancer progression (AS, androgen-sensitive; RAD, responsive to androgen-deprivation; and CR, castration-recurrent) cluster together. 13N, 15N, and 13R indicate the identity of each animal.

The LongSAGE libraries were hierarchically clustered and displayed as a phylogenetic tree. In most cases, LongSAGE libraries made from the same disease stage (AS, RAD, or CR) clustered together more closely than LongSAGE libraries made from the same biological replicate (mice 13N, 15N, or 13R; Figure 1). This suggests the captured transcriptomes were representative of disease stage with minimal influence from biological variation.

**Figure 2:**
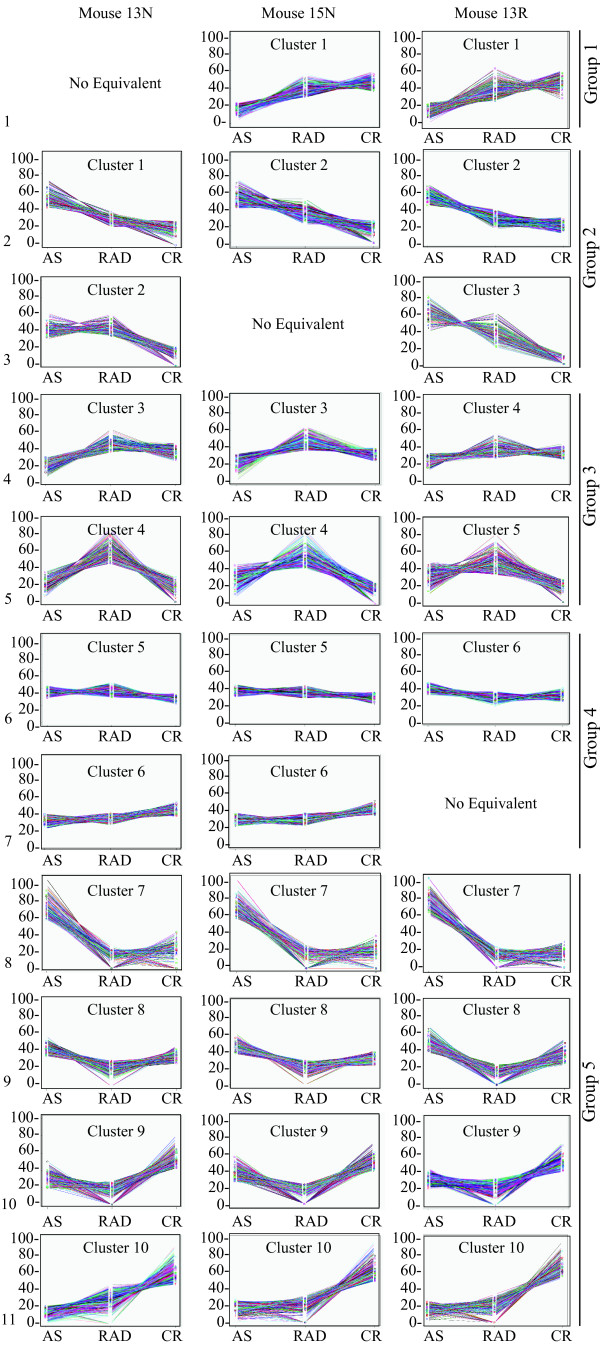
**K-means clustering of tag types with similar expression trends**. PoissonC with K = 10 (where K = number of clusters) was conducted over 100 iterations separately for each biological replicate (mice 13N, 15N, and 13R) and the results from the iterations were combined into consensus clusters shown here. Plotted on the x-axes are the long serial analysis of gene expression (LongSAGE) libraries representing different stages of prostate progression: AS, androgen-sensitive; RAD, responsive to androgen-deprivation; and CR, castration-recurrent. Plotted on the y-axes are the relative expression levels of each tag type, represented as a percentage of the total tag count (for a particular tag type) in all three LongSAGE libraries. Different colors represent different tag types. Each of the ten clusters for each biological replicate are labeled as such. 'No equivalent' indicates that a similar expression trend was not observed in the indicated biological replicate. Eleven expression patterns are evident in total and are labeled on the left. K-means clusters were amalgamated into five major expression trends: group 1, up during progression; group 2, down during progression; group 3, peak in the RAD stage; group 4, constant during progression; and group 5, valley in RAD stage.

Identification of groups of genes that behave similarly during progression of prostate cancer was conducted through K-means clustering of tags using the PoissonC algorithm [30]. For each biological replicate (mice 13N, 15N, or 13R), all tag types were clustered that had a combined count greater than ten in the three libraries representing disease stages (AS, RAD, and CR) and mapped unambiguously sense to a transcript in reference sequence (RefSeq; February 28^th^, 2008) [32] using DiscoverySpace4 software [33]. By plotting within cluster dispersion (i.e., intracluster variance) against a range of K (number of clusters; Additional file 1, Figure S2), we determined that ten clusters best embodied the expression patterns present in each biological replicate. This was decided based on the inflection point in the graph (Additional file 1, Figure S2), showing that after reaching K = 10, increasing the number of K did not substantially reduce the within cluster dispersion. K-means clustering was performed over 100 iterations, so that tags would be placed in clusters that best represent their expression trend. The most common clusters for each tag are displayed (Figure 2). In only three instances, there were similar clusters in just two of the three biological replicates. Consequently, consistent changes in gene expression during progression were represented in 11 patterns. Differences among expression patterns for each biological replicate may be explained by biological variation, the probability of sampling a given LongSAGE tag, and/or imperfections in K-means clustering (e.g, variance may not be a good measure of cluster scatter).

### Gene ontology enrichment analysis

We conducted Gene Ontology (GO) [34] enrichment analysis using Expression Analysis Systematic Explorer (EASE) [35] software to determine whether specific GO annotations were over-represented in the K-means clusters. Enrichment was defined by the EASE score (p-value ≤ 0.05) generated during comparison to all the other clusters in the biological replicate. This analysis was done for each biological replicate (3 mice: 13N, 15N, or 13R).

**Figure 3:**
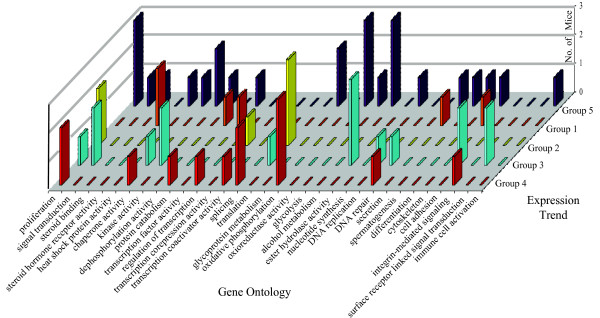
**Gene Ontology enrichments of the five major expression trends**. Plotted on the x-axis are Gene Ontology (GO) categories enriched in one or more of the five major expression trends. On the z-axis the five major expression trends correspond to Figure 2 and are: group 1, up during progression; group 2, down during progression; group 3, peak in the RAD stage; group 4, constant during progression; and group 5, valley in RAD stage. The y-axis displays the number of biological replicates (number of mice: 1, 2, or 3) exhibiting enrichment. The latter allows one to gauge the magnitude of the GO enrichment and confidence.

To enable visual differences between the 11 expression trends, the clusters were amalgamated into five major trends: group 1, up during progression; group 2, down during progression; group 3, peak in the RAD stage; group 4, constant during progression; and group 5, valley in RAD stage (Figure 2). To be consistent, the GO enrichment data was combined into five major trends which resulted in redundancy in GO terms. To simplify the GO enrichment data, similar terms were pooled into representative categories. Categorical gene ontology enrichments of the five major expression trends are shown in Figure 3. These data indicate that steroid binding, heat shock protein activity, de-phosphorylation activity, and glycolysis all decreased in the stage that was RAD, but increased again in the stage that was CR. Interestingly, steroid hormone receptor activity continues to increase throughout progression. Both of these expression trends were observed for genes with GO terms for transcription factor activity or secretion. The GO categories for genes with kinase activity and signal transduction displayed expression trends with peaks and valleys at the stage that was RAD. The levels of expression of genes involved in cell adhesion rose in the stage that was RAD, but dropped again in the stage that was CR.

Altogether, genes with functional categories that were enriched in expression trends may be consistent with the AR signaling pathway playing a role in progression of prostate cancer to castration-recurrence (Figure 3). For example, GO terms steroid binding, steroid hormone receptor activity, heat shock protein activity, chaperone activity, and kinase activity could represent the cytoplasmic events of AR signaling. GO terms transcription factor activity, regulation of transcription, transcription corepression activity, and transcription co-activator activity could represent the nuclear events of AR signaling. AR-mediated gene transcription may result in splicing and protein translation, to regulate general cellular processes such as proliferation (and related nucleotide synthesis, DNA replication, oxidative phosphorylation, oxioreductase activity, and glycolysis), secretion, and differentiation.

It should be noted, however, that both positive and negative regulators were represented in the GO enriched categories (Figure 3). Therefore, a more detailed analysis was required to determine if the pathways represented by the GO-enriched categories were promoted or inhibited during progression to CRPC. Moreover, many of the GO enrichments that were consistent with changes in the AR signaling pathway were generic, and could be applied to the other models of CRPC.

### Consistent differential gene expression associated with progression of prostate cancer

Pair-wise comparisons were made between LongSAGE libraries representing the transcriptomes of different stages (AS, RAD, and CR) of prostate cancer progression from the same biological replicate (3 mice: 13N, 15N, or 13R). Among all three biological replicates, the number of consistent statistically significant differentially expressed tag types were determined using the Audic and Claverie test statistic [36] at p ≤ 0.05, p ≤ 0.01, and p ≤ 0.001 (Table 2). The tags represented in Table 2 were included only if the associated expression trend was common among all three biological replicates. The Audic and Claverie statistical method is well-suited for LongSAGE data, because the method takes into account the sizes of the libraries and tag counts. Tag types were counted multiple times if they were over, or under-represented in more than one comparison. The number of tag types differentially expressed decreased by 57% as the stringency of the p-value increased from p ≤ 0.05 to 0.001.

**Table 2:** Number of tag types consistently and significantly differentially expressed among all three biological replicates and between conditions*

Comparison	Change	p ≤ 0.001	p ≤ 0.01	p ≤ 0.05
AS† vs. RAD‡	Up in RAD	21	44	83
	Down in RAD	68	105	149
	Total	89	149	232
				
RAD vs. CR§	Up in CR	24	45	89
	Down in CR	46	59	104
	Total	70	104	193
				
AS vs. CR	Up in CR	111	167	294
	Down in CR	127	168	256
	Total	238	335	550

* Statistics according to the Audic and Claverie test statistic

† AS, Androgen-sensitive

‡ RAD, Responsive to androgen-deprivation

§ CR, Castration-recurrent

Tag types consistently differentially expressed in pair-wise comparisons were mapped to RefSeq (March 4th, 2008). Tags that mapped anti-sense to genes, or mapped ambiguously to more than one gene were not included in the functional analysis. GO, Kyoto Encyclopedia of Genes and Genomes (KEGG; v45.0) [37] pathway, and SwissProt (v13.0) [38] keyword annotation enrichment analyses were conducted using EASE (v1.21; March 11^th^, 2008) and FatiGO (v3; March 11^th^, 2008) [39] (Table 3). This functional analysis revealed that the expression of genes involved in signaling increased during progression, but the expression of genes involved in protein synthesis decreased during progression. Cell communication increased in the stage that was RAD but leveled off in the stage that was CR. Carbohydrate, lipid and amino acid synthesis was steady in the RAD stage but increased in the CR stage. Lastly, glycolysis decreased in the RAD stage, but was re-expressed in the CR stage (Table 3).

**Table 3:** Top five enrichments of functional categories of tags consistently and significantly differentially expressed among all three biological replicates and between stages of prostate cancer*

Top 5 GO † categories	P-value ‡	Top 5 KEGG § annotations	P-value II	Top 5 SwissProt annotations	P-value II
AS vs. RAD: Up in RAD¶					

Cell communication	2.E-02	Stilbene, coumarine and lignin biosynthesis	1.E-02	Antioxidant	7.E-04
Extracellular	2.E-02	Butanoate metabolism	2.E-02	Cell adhesion	5.E-03
Extracellular matrix	2.E-02	2,4-Dichlorobenzoate degradation	2.E-02	Signal	6.E-03
Synaptic vesicle transport	3.E-02	Cell adhesion molecules (CAMs)	2.E-02	Fertilization	7.E-03
Synapse	4.E-02	Alkaloid biosynthesis II	5.E-02	Amyotrophic lateral sclerosis	7.E-03

AS vs. RAD: Down in RAD					

Glycolysis	3.E-05	Glycolysis/Gluconeogenesis	3.E-05	Glycolysis	3.E-07
Glucose catabolism	1.E-04	Ribosome	2.E-03	Pyrrolidone carboxylic acid	8.E-05
Hexose catabolism	1.E-04	Carbon fixation	3.E-03	Pyridoxal phosphate	2.E-04
Hexose metabolism	2.E-04	Fructose and mannose metabolism	2.E-02	Gluconeogenesis	3.E-04
Monosaccharide catabolism	2.E-04	Urea cycle and metabolism of amino groups	3.E-02	Coiled coil	5.E-03

RAD vs. CR: Up in CR					

Acid phosphatase activity	4.E-02	gamma-Hexachlorocyclohexane degradation	5.E-03	Lyase	2.E-03
Lyase activity**	7.E-02	Glycolysis/Gluconeogenesis	3.E-02	Immune response	5.E-03
Carbohydrate metabolism**	9.E-02	O-Glycan biosynthesis	5.E-02	Signal	6.E-03
Extracellular**	1.E-01	Ether lipid metabolism**	6.E-02	Glycolysis	7.E-03
Catabolism**	1.E-01	Phenylalanine, tyrosine and tryptophan biosynthesis**	6.E-02	Progressive external ophthalmoplegia	1.E-02

RAD vs. CR: Down in CR					

Cytosolic ribosome	2.E-09	Ribosome	2.E-11	Ribosomal protein	6.E-10
Large ribosomal subunit	1.E-07	Urea cycle and metabolism of amino groups	1.E-02	Ribonucleoprotein	3.E-08
Cytosol	2.E-07	Arginine and proline metabolism	4.E-02	Acetylation	1.E-05
Cytosolic large ribosomal subunit	2.E-07	Type II diabetes mellitus**	1.E-01	Elongation factor	1.E-03
Protein biosynthesis	2.E-07	Phenylalanine metabolism**	1.E-01	rRNA-binding	2.E-03

AS vs. CR: Up in CR					

Synapse	4.E-03	Butanoate metabolism	2.E-03	Glycoprotein	2.E-03
Extracellular	5.E-03	Ascorbate and aldarate metabolism	2.E-02	Vitamin C	7.E-03
Transition metal ion binding	7.E-03	Phenylalanine metabolism	2.E-02	Lipoprotein	1.E-02
Metal ion binding	2.E-02	Linoleic acid metabolism	2.E-02	Signal	1.E-02
Extracellular matrix	2.E-02	gamma-Hexachlorocyclohexane degradation	2.E-02	Heparin-binding	1.E-02

AS vs. CR: Down in CR					

Cytosolic ribosome	4.E-12	Ribosome	2.E-09	Acetylation	2.E-07
Biosynthesis	7.E-11	Carbon fixation	9.E-04	Ribosomal protein	1.E-06
Macromolecule biosynthesis	2.E-10	Glycolysis/Gluconeogenesis	3.E-03	Glycolysis	7.E-05
Protein biosynthesis	1.E-08	Glycosphingolipid biosynthesis - lactoseries	4.E-02	Ribonucleoprotein	8.E-05
Eukaryotic 43 S preinitiation complex	2.E-08	Glutamate metabolism**	8.E-02	Protein biosynthesis	1.E-04

* Statistics according to the Audic and Claverie test statistic (p ≤ 0.05)

† GO, Gene Ontology

‡ P-value represents the raw EASE (Expression Analysis Systematic Explorer) score

§ KEGG, Kyoto Encyclopedia of Genes and Genomes

II Unadjusted p-value was computed using FatiGO

¶ AS, androgen-sensitive; RAD, responsive to androgen-deprivation; CR, castration-recurrent

** Not statistically significant (p > 0.05)

Tag types differentially expressed between the RAD and CR stages of prostate cancer were of particular interest (Table 4). This is because these tags potentially represent markers for CRPC and/or are involved in the mechanisms of progression to CRPC. These 193 tag types (Table 2) were mapped to databases RefSeq (July 9^th^, 2007), Mammalian Gene Collection (MGC; July 9^th^, 2007) [40], or Ensembl Transcript or genome (v45.36d) [41]. Only 135 of the 193 tag types were relevant (Table 4) with 48 tag types that mapped ambiguously to more than one location in the *Homo Sapiens *transcriptome/genome, and another 10 tag types that mapped to *Mus musculus *transcriptome/genome. *Mus musculus *mappings may be an indication of minor contamination of the *in vivo *LNCaP Hollow Fiber model samples with host (mouse) RNA. These 135 tag types represented 114 candidate genes with 7 tag types that did not map to the genome, 5 tag types that mapped to unannotated genomic locations, and 9 genes that were associated with more than one tag type. Table 4 shows the LongSAGE tag sequences and tag counts per million tags in all nine libraries. Tags were sorted into groups based on expression trends. These trends are visually represented in Additional file 1, Figure S3. Mapping information was provided where available.

**Table 4:** Gene expression trends of LongSAGE tags that consistently and significantly altered expression in CR prostate cancer*

	13N			15N			13R					
				
	AS§	RADII	CR¶	AS	RAD	CR	AS	RAD	CR			
Tag Sequence	S1885	S1886	S1887	S1888	S1889	S1890	S1891	S1892	S1893	Trend‡	Gene**	Accession§§
TCTAGAGAACACTGTGC	12†	79	382	7	67	136	7	52	200	A	*ACPP*‡‡	NM_001099
TAATTTTTCTAAGGTGT	101	311	648	119	397	895	120	546	918	A	*C1ORF80*	ENSG00000186063
TGAGAGAGGCCAGAACA	8	39	150	4	39	144	7	33	95	A	N/A	Genomic
CTCATAAGGAAAGGTTA	637	952	1680	653	1170	1540	688	1620	1930	A	*RNF208*	BC090061
GATTTCTATTTGTTTTT	89	169	446	116	208	339	86	311	555	A	*SERINC5*	ENSG00000164300
GTTGGGAAGACGTCACC	426	571	742	273	417	741	262	363	495	A	*STEAP1*	NM_012449
GAGGATCACTTGAGGCC	191	299	449	134	189	589	187	203	314	B	*AMACR*‡‡	BC009471
TTGTTGATTGAAAATTT	219	197	528	273	197	479	232	391	586	B	*AMD1*‡‡	NM_001634
TTTGCTTTTGTTTTGTT	53	16	169	34	51	129	7	28	72	B	*AQP3*	NM_004925
GTTCGACTGCCCACCAG	45	28	101	52	47	122	34	42	106	B	*ASAH1*††	NM_177924
TAATAAACAGGTTTTTA	426	232	648	332	315	700	138	250	491	B	*ASAH1*‡‡	NM_177924
TCACAGCTGTGAAGATC	85	110	277	161	71	258	310	438	945	B	*BTG1*	NM_001731
AAAAGAGAAAGCACTTT	24	75	199	19	35	85	15	90	552	B	*CAMK2N1*	NM_018584
CAAAACAGGCAGCTGGT	4	71	169	15	83	162	37	75	268	B	*CAMK2N1*††	NM_018584
AGGAGGAAGAATGGACT	33	59	187	49	67	247	26	42	223	B	*CCNH*	NM_001239
TTTTAAAAATATAAAAT	89	83	243	97	130	269	64	170	382	B	*COMT*	NM_000754
GAATGAAATAAAAAATA	134	252	626	209	240	357	116	160	272	B	*DHRS7*	NM_016029
AAAGTGCATCCTTTCCC	118	146	318	153	220	394	288	231	646	B	*FGFRL1*	NM_001004356
AAACTGAATAAGGAGAA	24	51	236	19	51	438	19	146	283	B	*GALNT3*	NM_004482
TTTAAGGAAACATTTGA	4	4	75	4	4	81	0	0	57	B	*GALNT3*††	NM_004482
CCAACCGTGCTTGTACT	191	327	521	202	279	534	172	363	510	B	*GLO1*	NM_006708
GAGGGCCGGTGACATCT	300	378	1170	321	476	1230	254	447	1030	B	*H2AFJ*	NM_177925
TATCATTATTTTTACAA	57	63	161	67	63	181	75	94	181	B	*HSD17B4*	NM_000414
AATGCACTTATGTTTGC	16	8	64	22	16	77	19	28	98	B	N/A	No map
ACCTTCGCAGGGGAGAG	0	0	19	0	4	41	0	5	34	B	N/A	Genomic
ATAACCTGAAAGGAAAG	0	16	56	7	4	74	0	28	87	B	N/A	No map
GTGATGTGCACCTGTTG	0	0	38	4	0	30	0	5	45	B	N/A	No map
GTTTGGAGGTACTAAAG	20	43	94	34	87	169	34	90	234	B	N/A	Genomic
TTTTCAAAAATTGGAAA	0	35	180	7	4	59	0	19	61	B	N/A	No map
GAAAAATTTAAAGCTAA	394	397	569	433	598	788	853	862	1060	B	*NGFRAP1*	NM_206917
CAAATTCAGGGAGCACA	0	4	139	4	16	228	0	14	136	B	*OPRK1*	NM_000912
CTATTGTCTGAACTTGA	0	8	109	0	12	70	0	9	227	B	*OR51E2*	BC020768
ATGCTAATTATGGCAAT	4	12	75	4	8	74	0	5	57	B	*PCGEM1*	NR_002769
CAGAAAGCATCCCTCAC	4	43	195	0	16	111	7	33	264	B	*PLA2G2A*‡‡	NM_000300
TAATTTTAGTGCTTTGA	16	75	154	37	59	162	4	57	132	B	*PTGFR*	NM_000959
TTGTTTGTAAATAGAAT	0	12	94	0	4	162	0	14	72	B	*QKI*	NM_206853
TAAACACTGTAAAATCC	0	4	75	0	4	66	0	0	42	B	*QKI*††	NM_206853
AGCAGATCAGGACACTT	20	35	112	15	16	140	15	42	98	B	*S100A10*	NM_002966
CTGCCATAACTTAGATT	37	55	161	93	63	192	56	99	264	B	*SBDS*	NM_016038
TGGCTGAGTTTATTTTT	20	24	79	41	8	96	4	42	147	B	*SFRS2B*	NM_032102
GAAGATTAATGAGGGAA	126	142	277	108	130	402	101	188	325	B	*SNX3*	NM_003795
ATGGTACTAAATGTTTT	16	47	124	37	28	88	11	19	76	B	*SPIRE1*	NM_020148
TATATATTAAGTAGCCG	45	39	101	45	75	133	41	75	178	B	*STEAP2*‡‡	NM_152999
CAACAATATATGCTTTA	24	32	82	75	32	136	26	99	212	B	*STEAP2*††	NM_152999
TTTCATTGCCTGAATAA	24	43	150	34	59	114	22	61	178	B	*TACC1*‡‡	NM_006283
TTGGCCAGTCTGCTTTC	8	16	67	4	4	77	0	5	38	B	*TMEM30A*	ENSG00000112697
ATATCACTTCTTCTAGA	12	4	26	7	4	26	0	52	140	C	*ADAM2*‡‡	NM_001464
ATGTGTGTTGTATTTTA	812	338	768	1010	315	1020	269	702	865	C	*BNIP3*	NM_004052
CCACGTTCCACAGTTGC	601	291	599	530	346	700	381	339	559	C	*ENO2*	NM_001975
CTGATCTGTGTTTCCTC	16	0	26	0	4	41	19	0	34	C	*HLA-B*	BC013187
AGCCCTACAAACAACTA	382	441	596	508	456	619	400	631	1010	C	*MT-ND3*	ENSG00000198840
ATATTTTCTTTGTGGAA	20	12	90	7	0	48	4	0	23	C	N/A	No map
CAAGCATCCCCGTTCCA	2400	2130	2440	2730	1720	2250	1020	2010	2340	C	N/A	ENSG00000211459
GTTGTAAAATAAACTTT	118	83	172	228	87	247	112	203	378	C	N/A	Genoic
TTGGATTTCCAAAGCAG	12	0	19	0	0	33	0	0	26	C	N/A	Genomic
TCTTTTAGCCAATTCAG	138	181	420	381	326	468	389	334	457	C	*NKX3-1*††	NM_006167
TGATTGCCCTTTCATAT	73	39	86	86	39	107	108	99	181	C	*P4HA1*	NM_000917
GTAACAAGCTCTGGTAT	28	16	56	49	24	66	11	19	72	C	*PJA2*	NM_014819
ACAGTGCTTGCATCCTA	85	75	139	108	98	203	101	118	196	C	*PPP2CB*	NM_004156
AGGCGAGATCAATCCCT	57	39	101	37	24	122	131	66	268	C	*PSMA7*	NM_002792
TATTTTGTATTTATTTT	73	59	180	93	51	111	22	94	253	C	*SLC25A4*	NM_001151
TTATGGATCTCTCTGCG	1050	1260	1820	1140	1300	2260	1990	1010	1530	C	*SPON2*	NM_012445
CAGTTCTCTGTGAAATC	767	515	1060	855	503	914	467	608	1200	C	*TMEM66*	NM_016127
AAATAAATAATGGAGGA	138	59	255	82	118	284	165	90	159	C	*TRPM8*	NM_024080
ATGTTTAATTTTGCACA	61	87	154	157	59	195	217	85	344	C	*WDR45L*	NM_019613
GGGCCCCAAAGCACTGC	861	543	1180	1020	657	1590	1240	739	937	E	*C19orf48*	NM_199249
TCCCCGTGGCTGTGGGG	1670	1390	2290	1740	1410	1720	3370	970	1180	E	*DHCR24*‡‡	BC004375
GCATCTGTTTACATTTA	487	201	345	444	208	468	684	226	423	E	*ELOVL5*	NM_021814
GAAATTAGGGAAGCCTT	317	153	311	310	181	542	359	193	298	E	*ENDOD1*	XM_290546
GGATGGGGATGAAGTAA	2780	1160	4780	2950	1350	3620	2930	1230	1890	E	*KLK3*‡‡	NM_001648
TGAAAAGCTTAATAAAT	313	142	322	474	181	332	273	179	314	E	*TPD52*	NM_001025252
GTTGTGGTTAATCTGGT	1770	634	1270	1800	806	1190	2480	659	960	F	*B2M*	NM_004048
GAAACAAGATGAAATTC	4380	1170	2260	5300	1110	2720	3750	2220	2830	F	*PGK1*	NM_000291
AGCACCTCCAGCTGTAC	2150	1130	648	2060	1560	939	1560	1200	722	G	*EEF2*	NM_001961
GCACAAGAAGATTAAAA	536	228	124	762	425	195	838	278	174	G	*GAS5*	NR_002578
CCGCTGCGTGAGGGCAG	451	169	56	429	197	44	516	94	0	G	*HES6*	NM_018645
GCCCAGGTCACCCACCC	585	55	4	519	79	7	456	66	0	G	*LOC644844*	XM_927939
ATGCAGCCATATGGAAG	2650	386	82	2470	216	129	1210	259	98	G	*ODC1*	NM_002539
CGCTGGTTCCAGCAGAA	1420	811	479	1250	959	553	800	589	374	G	*RPL11*	NM_000975
AAGACAGTGGCTGGCGG	2650	1730	1220	2460	1860	1350	2120	1630	1270	G	*RPL37A*‡‡	NM_000998
TTCTTGTGGCGCTTCTC	925	543	217	1030	708	273	1130	419	306	G	*RPS11*††	NM_001015
GGTGAGACACTCCAGTA	463	252	165	485	346	192	363	245	159	G	*SLC25A6*	NM_001636
AGGTTTTGCCTCATTCC	982	515	281	1200	491	243	688	782	166	H	*ABHD2*	NM_007011
TGAAGGAGCCGTCTCCA	317	272	187	392	295	199	366	259	140	H	*ATP5G2*	NM_001002031
CTCAGCAGATCCAAGAG	191	185	67	254	232	66	142	231	79	H	*C17orf45*	NM_152350
CTGTGACACAGCTTGCC	308	397	172	209	307	125	295	226	110	H	*CCT2*	NM_006431
TCTGCACCTCCGCTTGC	495	606	277	426	570	276	366	471	204	H	*EEF1A2*	NM_001958
GCCCAAGGACCCCCTGC	114	114	38	138	98	41	101	42	4	H	*FLNA*‡‡	NM_001456
TTATGGGATCTCAACGA	564	425	180	642	452	317	430	490	253	H	*GNB2L1*	NM_006098
TCTGCAAAGGAGAAGTC	81	102	38	105	87	26	165	80	30	H	*HMGB2*	NM_002129
CTTGTGAACTGCACAAC	268	228	124	231	177	103	273	160	57	H	*HN1*	NM_016185
TCTGAAGTTTGCCCCAG	313	291	150	254	299	155	187	226	72	H	*MAOA*	NM_000240
TTAATTGATAGAATAAA	483	350	199	422	287	103	273	235	83	H	*MAOA*	NM_000240
GGCAGCCAGAGCTCCAA	1200	1260	420	1050	672	350	681	819	23	H	*MARCKSL1*	NM_023009
CCCTGCCTTGTCCCTCT	353	240	112	310	263	107	176	193	102	H	*MDK*	NM_001012334
CTGTGGATGTGTCCCCC	649	476	169	459	389	214	430	297	117	H	N/A	No map
CTCCTCACCTGTATTTT	1120	771	262	1220	979	313	666	730	261	H	*RPL13A*‡‡	NM_012423
GCAGCCATCCGCAGGGC	1980	1770	809	2300	1730	928	2150	1570	1020	H	*RPL28*	NM_000991
GGATTTGGCCTTTTTGA	3470	2070	1370	4170	2910	1540	2800	2870	2500	H	*RPLP2*‡‡	NM_001004
TCTGTACACCTGTCCCC	2320	1670	850	1930	1880	825	2130	1490	1120	H	*RPS11*	NM_001015
GCTTTTAAGGATACCGG	1510	1050	626	1860	1120	593	1550	1550	960	H	*RPS20*‡‡	NM_001023
CCCCAGCCAGTCCCCAC	921	519	281	788	664	357	1100	438	291	H	*RPS3*	NM_001005
CCCCCAATGCTGAGGCC	89	138	26	90	94	30	90	80	30	H	*SF3A2*	NM_007165
GCCGCCATCTCCGAGAG	195	102	30	168	118	55	172	108	30	H	*TKT*	NM_001064
GGCCATCTCTTCCTCAG	349	307	202	317	346	173	277	254	121	H	*YWHAQ*	NM_006826
AGGCTGTGTTCCTCCGT	16	39	11	34	67	22	26	38	8	I	*ACY1*	NM_000666
TGCCTCTGCGGGGCAGG	446	649	427	399	664	424	501	462	317	I	*CD151*	NM_004357
GGCACAGTAAAGGTGGC	175	216	142	332	350	173	456	316	204	I	*CUEDC2*	NM_024040
TCACACAGTGCCTGTCG	49	71	7	30	47	15	34	66	4	I	*CXCR7*	NM_001047841
TGTGAGGGAAGCTGCTT	53	87	15	67	102	52	52	90	42	I	*FKBP10*	BC016467
TGCTTTGCTTCATTCTG	28	63	26	22	79	26	49	118	61	I	*GRB10*	NM_005311
GTACTGTATGCTTGCCA	170	212	82	134	153	88	123	188	113	I	*KPNB1*‡‡	NM_002265
GTGGCAGTGGCCAGTTG	106	193	97	123	173	96	94	137	76	I	N/A	ENSG00000138744
GGGGAGCCCCGGGCCCG	61	63	26	30	51	18	34	57	0	I	*NAT14*	NM_020378
TGTTCAGGACCCTCCCT	28	67	26	60	63	26	60	28	0	I	*NELF*	NM_015537
TTTTCCTGGGGATCCTC	41	130	15	37	87	33	56	104	45	I	PCOTH	NM_001014442
GAAACCCGGTAGTCTAG	41	75	4	37	75	26	52	151	30	I	*PLCB4*	NM_000933
GTCTGACCCCAGGCCCC	126	205	82	119	193	103	157	179	38	I	*PPP2R1A*	NM_014225
GGCCCGAGTTACTTTTC	231	150	75	161	232	136	142	160	45	I	*RPL35A*††	NM_000996
GTTCGTGCCAAATTCCG	881	696	390	1100	712	523	497	782	461	I	*RPL35A*‡‡	NM_000996
TTACCATATCAAGCTGA	877	535	311	1130	598	405	636	791	578	I	*RPL39*‡‡	NM_001000
GCTGCAGCACAAGCGGC	268	244	127	45	216	125	157	71	11	I	*RPS18*††	NM_022551
AGCTCTTGGAGGCACCA	203	319	206	142	421	243	269	259	162	I	*SELENBP1*	NM_003944
TGCTGGTGTGTAAGGGG	69	102	45	82	87	37	105	75	30	I	*SH3BP5L*	NM_030645
GAGAGTAACAGGCCTGC	191	150	71	112	181	111	108	165	64	I	*SYNC1*	NM_030786
CTGAAAACCACTCAAAC	394	508	225	306	547	236	310	381	200	I	*TFPI*	NM_006287
TAAAAAAGGTTTCATCC	183	248	127	86	130	66	142	268	87	I	*TFPI*	NM_006287
CTCCCTCCTCTCCTACC	28	32	4	30	39	7	71	24	0	I	*TK1*	NM_003258
CATTTTCTAATTTTGTG	544	744	236	407	771	181	288	664	185	J	N/A	No map
TGATTTCACTTCCACTC	3480	5260	3910	3700	6110	3590	3040	5960	2600	K	*MT-CO3*	ENSG00000198938
TTTCTGTCTGGGGAAGG	130	236	82	123	201	111	101	188	113	K	*PIK3CD*	NM_005026
GCCGCTACTTCAGGAGC	256	370	199	224	330	169	142	316	38	K	*RAMP1*	NM_005855
ATGGTTACACTTTTGGT	93	161	94	75	208	118	60	226	95	K	*UTX*	NM_021140
CACTACTCACCAGACGC	2820	3900	3020	2740	4290	2440	2620	3120	1260	K	*VPS13B*††	ENSG00000132549
CTAAGACTTCACCAGTC	7120	11000	9730	6390	10900	8330	3610	8870	7850	L	N/A	ENSG00000210082

* Statistics according to the Audic and Claverie test statistic (p ≤ 0.05)

† Tag count per 1 million = (observed tag count/total tags in the library) × 1,000,000

‡ Trends are visually represented from A to P in Additional file 1, Figure S3. In addition to p-value considerations, significantly different trends were also required to display uniform directions of change in each biological replicate.

§ AS, Androgen-sensitive

II RAD, Responsive to androgen-deprivation

¶ CR, Castration-recurrent

** Human Genome Nomenclature Committee (HGNC)-approved gene names were used when possible. Non-HGNC-approved gene names were not italicized.

†† Tag maps antisense to gene

‡‡ Gene is known to display this expression trend in castration-recurrence

§§ Accession numbers were displayed following the priority (where available): RefSeq > Mammalian Gene Collection > Ensembl Gene. If the tag mapped to more than one transcript variant of the same gene, the accession number of the lowest numerical transcript variant was displayed.

We cross-referenced these 114 candidate genes with 28 papers that report global gene expression analyses on tissue samples from men with 'castration-recurrent', 'androgen independent,' 'hormone refractory,' 'androgen-ablation resistant,' 'relapsed,' or 'recurrent' prostate cancer, or animal models of castration-recurrence [42-69]. The candidate genes were identified with HUGO Gene Nomenclature Committee (HGNC) approved gene names, aliases, descriptions, and accession numbers. The gene expression trends of 18 genes of 114 genes were previously associated with CRPC. These genes were: *ACPP, ADAM2, AMACR, AMD1, ASAH1, DHCR24, FLNA, KLK3, KPNB1, PLA2G2A, RPL13A, RPL35A, RPL37A, RPL39, RPLP2, RPS20, STEAP2*, and *TACC *(Table 4). To our knowledge, the gene expression trends of the remaining 96 genes have never before been associated with CRPC (Tables 4 &5).

A literature search helped to gauge the potential of these 96 genes to be novel biomarkers or therapeutic targets of CRPC. The results of this literature search are presented in Table 5. We found 31 genes that encode for protein products that are known, or predicted, to be plasma membrane bound or secreted extracellularly (Bioinformatic Harvester). These genes were: *ABHD2, AQP3, B2 M, C19orf48, CD151, CXCR7, DHRS7, ELOVL5, ENDOD1, ENO2, FGFRL1, GNB2L1, GRB10, HLA-B, MARCKSL1, MDK, NAT14, NELF, OPRK1, OR51E2, PLCB4, PTGFR, RAMP1, S100A10, SPON2, STEAP1, TFPI, TMEM30A, TMEM66, TRPM8*, and *VPS13B*. Secretion of a protein could facilitate detection of the putative biomarkers in blood, urine, or biopsy sample. Twenty-one of the candidate genes are known to alter their levels of expression in response to androgen. These genes were: *ABHD2, B2 M, BTG1, C19orf48, CAMK2N1, CXCR7, EEF1A2, ELOVL5, ENDOD1, HSD17B4, MAOA, MDK, NKX3-1, ODC1, P4HA1, PCGEM1, PGK1, SELENBP1, TMEM66, TPD52*, and *TRPM8 *[9,22,70-81]. Genes regulated by androgen may be helpful in determining the activation status of AR in CRPC. Enriched expression of a protein in prostate tissue could be indicative of whether a tumor is of prostatic origin. Eight of these 96 genes are known to be over-represented in prostate tissue [75,82-85]. These genes were: *ELOVL5, NKX3-1, PCGEM1, PCOTH, RAMP1, SPON2, STEAP1*, and *TPD52*. Twenty-six genes (*ABHD2, BNIP3, EEF1A2, ELOVL5, GALNT3, GLO1*, *HSD17B4, MARCKSL1, MDK, NGFRAP1, ODC1, OR51E2, PCGEM1*, PCOTH, *PGK1, PP2CB, PSMA7, RAMP1, RPS18, SELENBP1, SLC25A4, SLC25A6, SPON2, STEAP1, TPD52*, and *TRPM8*) have known associations to prostate cancer [57,82,86-102]. Six genes (*C1orf80, CAMK2N1, GLO1, MAOA, PGK1*, and *SNX3*) have been linked to high Gleason grade [58,103,104], and twelve genes (*B2 M, CAMK2N1, CD151, COMT, GALNT3, GLO1, ODC1, PCGEM1*, PCOTH, *SBDS, TMEM30A*, and *TPD52*) have been implicated in the 'progression' of prostate cancer [58,82], and 15 more genes (*CD151, CXCR7, DHRS7, GNB2L1, HES6, HN1, NKX3-1, PGK1, PIK3CD, RPL11, RPS11, SF3A2, TK1, TPD52*, and *VPS13B*) in the metastasis of prostate cancer [105,106].

**Table 5:** Characteristics of genes with novel association to castration-recurrence *in vivo*

				Associated with									Associated with				
									
Gene*	S or PM†	Reg. by A‡	Spec. to P§	CaPII	GG¶	Prog.**	Mets††	CR‡‡	Gene	S or PM	Reg. by A	Spec. to P	CaP	GG	**Prog**.	Mets	CR
*ABHD2*	PM	Y↑	-	Y↑	-	-	-	-	*NKX3-1*	-	Y↑	Y	-	-	-	Y	-
*ACY1*	-	-	-	-	-	-	-	-	*ODC1*	-	Y↑	-	Y↑	-	Y↓	-	Y↑
*AQP3*	PM	-	-	-	-	-	-	-	*OPRK1*	PM	-	-	-	-	-	-	-
*ATP5G2*	-	-	-	-	-	-	-	-	*OR51E2*	PM	-	-	Y↑	-	-	-	-
*B2M*	S&PM	Y↑	-	-	-	Y↑	-	Y↓	*P4HA1*	-	Y	-	-	-	-	-	-
*BNIP3*	-	-	-	Y↓	-	-	-	-	*PCGEM1*	-	Y↑	Y	Y↑	-	Y↑	-	-
*BTG1*	-	Y↓	-	-	-	-	-	-	PCOTH	-	-	Y	Y↑	-	Y↑	-	-
*C17orf45*	-	-	-	-	-	-	-	-	*PGK1*	-	Y↑	-	Y↓	Y↑	-	Y ↑↓§§	-
C19orf48	S	Y↑	-	-	-	-	-	-	PIK3CD	-	-	-	-	-	-	Y↑	Y↑
C1orf80	-	-	-	-	Y↑	-	-	-	PJA2	-	-	-	-	-	-	-	-
CAMK2N1	-	Y↓	-	-	Y↑	Y↑	-	-	PLCB4	PM	-	-	-	-	-	-	-
CCNH	-	-	-	-	-	-	-	-	PPP2CB	-	-	-	Y↓	-	-	-	-
CCT2	-	-	-	-	-	-	-	-	PPP2R1A	-	-	-	-	-	-	-	-
CD151	PM	-	-	-	-	Y↑	Y↑	-	PSMA7	-	-	-	Y↓	-	-	-	-
COMT	-	-	-	-	-	Y↓	-	-	PTGFR	PM	-	-	-	-	-	-	-
CUEDC2	-	-	-	-	-	-	-	-	QKI	-	-	-	-	-	-	-	-
CXCR7	PM	Y↓	-	-	-	-	Y↑	Y↑	RAMP1	PM	-	Y	Y↑	-	-	-	-
DHRS7	PM	-	-	-	-	-	Y↓	-	RNF208	-	-	-	-	-	-	-	-
EEF1A2	-	Y↑	-	Y↑	-	-	-	-	RPL11	-	-	-	-	-	-	Y↓	-
EEF2	-	-	-	-	-	-	-	-	RPL28	-	-	-	-	-	-	-	-
ELOVL5	PM	Y	Y	Y↓	-	-	-	-	RPS11	-	-	-	-	-	-	Y↓	-
*ENDOD1*	S	Y↑	-	-	-	-	-	-	*RPS18*	-	-	-	Y↑	-	-	-	-
*ENO2*	PM	-	-	-	-	-	-	-	*RPS3*	-	-	-	-	-	-	-	-
ENSG00000210082	-	-	-	-	-	-	-	-	*S100A10*	PM	-	-	-	-	-	-	-
ENSG00000211459	-	-	-	-	-	-	-	-	*SBDS*	-	-	-	-	-	Y↑	-	-
*FGFRL1*	PM	-	-	-	-	-	-	-	*SELENBP1*	-	Y↓	-	Y↓	-	-	-	-
*FKBP10*	-	-	-	-	-	-	-	-	*SERINC5*	-	-	-	-	-	-	-	-
*GALNT3*	-	-	-	Y↑	-	Y↓	-	-	*SF3A2*	-	-	-	-	-	-	Y↑	-
GAS5	-	-	-	-	-	-	-	-	SFRS2B	-	-	-	-	-	-	-	-
GLO1	-	-	-	Y↑	Y↑	Y↑	-	-	SH3BP5L	-	-	-	-	-	-	-	-
GNB2L1	PM	-	-	-	-	-	Y↑	-	SLC25A4	-	-	-	Y↑	-	-	-	-
GRB10	PM	-	-	-	-	-	-	-	SLC25A6	-	-	-	Y↑	-	-	-	-
H2AFJ	-	-	-	-	-	-	-	-	SNX3	-	-	-	-	Y↑	-	-	-
HES6	-	-	-	-	-	-	Y↑	Y↑	*SPIRE1*	-	-	-	-	-	-	-	-
*HLA-B*	PM	-	-	-	-	-	-	-	*SPON2*	S	-	Y	Y↑	-	-	-	-
*HMGB2*	-	-	-	-	-	-	-	Y↑	*STEAP1*	PM	-	Y	Y↑	-	-	-	-
*HN1*	-	-	-	-	-	-	Y↑	-	*SYNC1*	-	-	-	-	-	-	-	-
*HSD17B4*	-	Y↑	-	Y↑	-	-	-	-	*TFPI*	S	-	-	-	-	-	-	-
LOC644844	-	-	-	-	-	-	-	-	*TK1*	-	-	-	-	-	-	Y↑	-
*MAOA*	-	Y	-	-	Y↑	-	-	-	*TKT*	-	-	-	-	-	-	-	-
*MARCKSL1*	PM	-	-	Y↑	-	-	-	-	*TMEM30A*	S&PM	-	-	-	-	Y↑	-	-
*MDK*	S&PM	Y↓	-	Y↑	-	-	-	Y↑	*TMEM66*	S&PM	Y↑	-	-	-	-	-	-
*MT-CO3*	-	-	-	-	-	-	-	-	*TPD52*	-	Y↑	Y	Y↑	-	Y↑	Y↓	-
*MT-ND3*	-	-	-	-	-	-	-	-	*TRPM8*	PM	Y↑	-	Y↑	-	-	-	Y↓
*NAAA*	-	-	-	-	-	-	-	Y↑	*UTX*	-	-	-	-	-	-	-	-
*NAT14*	PM	-	-	-	-	-	-	-	*VPS13B*	PM	-	-	-	-	-	Y↑	-
*NELF*	PM	-	-	-	-	-	-	-	*WDR45L*	-	-	-	-	-	-	-	-
*NGFRAP1*	-	-	-	Y↓	-	-	-	-	*YWHAQ*	-	-	-	-	-	-	-	-

* Human Genome Nomenclature Committee (HGNC)-approved gene names were used when possible. Non-HGNC-approved gene names were not italicized.

† S or PM, gene product is thought to be secreted (S) or localize to the plasma membrane (PM)

‡ Reg. by A, gene expression changes in response to androgen in prostate cells

§ Spec. to P, gene expression is specific to- or enriched in- prostate tissue compared to other tissues

II CaP, gene is differentially expressed in prostate cancer compared to normal, benign prostatic hyperplasia, or prostatic intraepithelial neoplasia

¶ GG, gene is differentially expressed in higher Gleason grade tissue versus lower Gleason grade tissue

** Prog., gene expression correlates with late-stage prostate cancer or is a risk factor that predicts progression

†† Mets, gene expression is associated with prostate cancer metastasis in human samples or *in vivo *models

‡‡ CR, gene is associated with castration-recurrent prostate cancer in human tissue or *in vivo *models, but exhibits an opposite trend of this report

§§ Y, yes; ↑, high gene expression; ↓, low gene expression

### Novel CR-associated genes identify both clinical samples of CRPC and clinical metastasis of prostate cancer

The expression of novel CR-associated genes were validated in publically available, independent sample sets representing different stages of prostate cancer progression (Gene Expression Omnibus accession numbers: GDS1390 and GDS1439). Dataset GDS1390 includes expression data of ten AS prostate tissues, and ten CRPC tissues from Affymetrix U133A arrays [47]. Dataset GDS1439 includes expression data of six benign prostate tissues, seven localized prostate cancer tissues, and seven metastatic prostate cancer tissues from Affymetrix U133 2.0 arrays [97].

**Figure 4:**
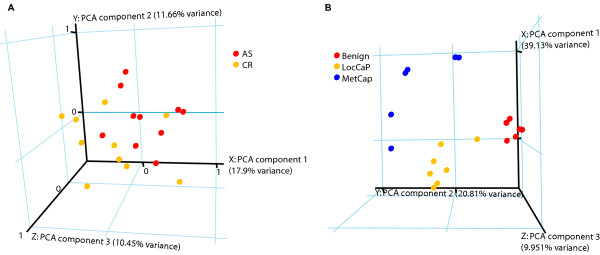
**Principle component analyses of clinical samples**. *A*, Principle component analysis based on the expression of novel CR-associated genes in the downloaded dataset GDS1390 clustered the AS and CR clinical samples into two groups. *B*, Principle component analysis based on the expression of novel CR-associated genes in the downloaded dataset GDS1439 clustered the clinical samples (benign prostate tissue, benign; localized prostate cancer, Loc CaP; and metastatic prostate cancer, Met CaP) into three groups.

Unsupervised principal component analysis based on the largest three principal components revealed separate clustering of tumor samples representing AS and CR stages of cancer progression, with the exception of two CR samples and one AS sample (Figure 4a).

Metastatic prostate cancer is expected to have a more progressive phenotype and is associated with hormonal progression. Therefore, the gene expression signature obtained from the study of hormonal progression may be common to that observed in clinical metastases. Unsupervised principal component analysis based on the largest three principal components revealed separate clustering of not only benign and malignant, but also localized and metastatic tissue samples (Figure 4b).

## Discussion

Genes that change levels of expression during hormonal progression may be indicative of the mechanisms involved in CRPC. Here we provide the most comprehensive gene expression analysis to date of prostate cancer with approximately 3 million long tags sequenced using *in vivo *samples of biological replicates at various stages of hormonal progression to improve over the previous libraries that are approximately 70,000 short tags or less. Previous large-scale gene expression analyses have been performed with tissue samples from men with advanced prostate cancer [42-58], and animal or xenograft models of CRPC [59-69]. Most of these previous studies compared differential expression between CRPC samples with the primary samples obtained before androgen ablation. This experimental design cannot distinguish changes in gene expression that are a direct response to androgen ablation, or from changes in proliferation/survival that have been obtained as the prostate cancer cells progress to more a more advanced phenotype. Here we are the first to apply an *in vivo *model of hormonal progression to compare gene expression between serial samples of prostate cancer before (AS), and after androgen ablation therapy (RAD) as well as when the cells become CR. This model is the LNCaP Hollow Fiber model [21] which has genomic similarity with clinical prostate cancer [23] and mimics the hormonal progression observed clinically in response to host castration as measured by levels of expression of PSA and cell proliferation. Immediately prior to castration, when the cells are AS, PSA levels are elevated and the LNCaP cells proliferate. A few days following castration, when the cells are RAD, PSA levels drop and the LNCaP cells cease to proliferate, but do not apoptose in this model. Approximately 10 weeks following castration, when the cells are CR, PSA levels rise and the LNCaP cells proliferate in the absence of androgen. This model overcomes some limitations in other studies using xenografts that include host contamination of prostate cancer cells. The hollow fibers prevent infiltration of host cells into the fiber thereby allowing retrieval of pure populations of prostate cells from within the fiber. The other important benefit of the fiber model is the ability to examine progression of cells to CRPC at various stages within the same host mouse over time, because the retrieval of a subset of fibers entails only minor surgery. The power to evaluate progression using serial samples from the same mouse minimizes biological variation to enhance the gene expression analyses. However, limitations of this model include the lack of cell-cell contact with stroma cells, and lack of heterogeneity in tumors. Typically, these features would allow paracrine interactions as expected in clinical situations. Consistent with the reported clinical relevance of this model [23], here principal component analysis based on the expression of these novel genes identified by LongSAGE, clustered the clinical samples of CRPC separately from the androgen-dependent samples. Principal component analysis based on the expression of these genes also revealed separate clustering of the different stages of tumor samples and also showed separate clustering of the benign samples from the prostate cancer samples. Therefore, some common changes in gene expression profile may lead to the survival and proliferation of prostate cancer and contribute to both distant metastasis and hormonal progression. We used this LNCaP atlas to identify changes in gene expression that may provide clues of underlying mechanisms resulting in CRPC. Suggested models of CRPC involve: the AR; steroid synthesis and metabolism; neuroendocrine prostate cancer cells; and/or an imbalance of cell growth and cell death.

### Androgen receptor (AR)

#### Transcriptional activity of AR

The AR is suspected to continue to play an important role in the hormonal progression of prostate cancer. The AR is a ligand-activated transcription factor with its activity altered by changes in its level of expression or by interactions with other proteins. Here, we identified changes in expression of some known or suspected modifier of transcriptional activity of the ARin CRPC versus RAD such as Cyclin H (*CCNH*) [107], proteasome macropain subunit alpha type 7 (*PSMA7*) [108], CUE-domain-containing-2 (*CUEDC2*) [109], filamin A (*FLNA*) [110], and high mobility group box 2 (*HMGB2*) [111]. *CCNH *and *PSMA7 *displayed increased levels of expression, while *CUEDC2, FLNA*, and *HMGB2 *displayed decreased levels of expression in CR. The expression trends of *CCNH, CUEDC2, FLNA*, and *PSMA7 *in CRPC may result in increased AR signaling through mechanisms involving protein-protein interactions or altering levels of expression of AR. CCNH protein is a component of the cyclin-dependent activating kinase (CAK). CAK interacts with the AR and increases its transcriptional activity [107]. Over-expression of the proteosome subunit PSMA7 promotes AR transactivation of a PSA-luciferase reporter [108]. A fragment of the protein product of *FLNA *negatively regulates transcription by AR through a physical interaction with the hinge region [110]. CUEDC2 protein promotes the degradation of progesterone and estrogen receptors [109]. These steroid receptors are highly related to the AR, indicating a possible role for CUEDC2 in AR degradation. Thus decreased expression of *FLNA *or *CUEDC2 *could result in increased activity of the AR. Decreased expression of *HMGB2 *in CRPC is predicted to decrease expression of at least a subset of androgen-regulated genes that contain palindromic AREs [111]. Here, genes known to be regulated by androgen were enriched in expression trend categories with a peak or valley at the RAD stage of prostate cancer progression. Specifically, 8 of the 13 tags (62%) exhibiting these expression trends 'E', 'F', 'J', 'K', or 'L' represented known androgen-regulated genes, in contrast to only 22 of the remaining 122 tags (18%; Tables 4 &5). Overall, this data supports increased AR activity in CRPC, which is consistent with re-expression of androgen-regulated genes as previously reported [68] and similarity of expression of androgen regulated genes between CRPC and prostate cancer before androgen ablation [23].

#### Steroid synthesis and metabolism

In addition to changes in expression of AR or interacting proteins altering the transcriptional activity of the AR, recent suggestion of sufficient levels of residual androgen in CRPC provides support for an active ligand-bound receptor [112]. The AR may become re-activated in CRPC due to the presence of androgen that may be synthesized by the prostate *de novo *[4] or through the conversion of adrenal androgens. Here, the expression of 5 genes known to function in steroid synthesis or metabolism were significantly differentially expressed in CRPC versus RAD. They are 24-dehydrocholesterol reductase (*DHCR24*) [113], dehydrogenase/reductase SDR-family member 7 (*DHRS7*) [114], elongation of long chain fatty acids family member 5 (*ELOVL5*) [115,116], hydroxysteroid (17-beta) dehydrogenase 4 (*HSD17B4*) [117], and opioid receptor kappa 1 (*OPRK1*) [118]. Increased levels of expression of these genes may be indicative of the influence of adrenal androgens, or the local synthesis of androgen, to reactivate the AR to promote the progression of prostate cancer in the absence of testicular androgens.

### Neuroendocrine

Androgen-deprivation induces neuroendocrine differentiation of prostate cancer. Here, the expression of 8 genes that are associated with neuroendocrine cells were significantly differentially expressed in CRPC versus RAD. They either responded to androgen ablation such as hairy and enhancer of split 6 (*HES6*) [119], karyopherin/importin beta 1 (*KPNB1*) [120], monoamine oxidase A (*MAOA*)[121], and receptor (calcitonin) activity modifying protein 1 (*RAMP1*) [122]], or were increased expressed in CRPC such as *ENO2 *[122], *OPRK1 *[118], S100 calcium binding protein A10 (*S100A10*) [123], and transient receptor potential cation channel subfamily M member 8 (*TRPM8*) [124].

### Proliferation and Cell survival

The gene expression trends of *GAS5 *[125], *GNB2L1 *[126], *MT-ND3, NKX3-1 *[127], *PCGEM1 *[128], *PTGFR *[129], *STEAP1 *[130], and *TMEM30A *[131] were in agreement with the presence of proliferating cells in CRPC. Of particular interest is that we observed a transcript anti-sense to *NKX3-1*, a tumor suppressor, highly expressed in the stages of cancer progression that were AS and CR, but not RAD. Anti-sense transcription may hinder gene expression from the opposing strand, and therefore, represents a novel mechanism by which *NKX3-1 *expression may be silenced. There were also some inconsistencies including the expression trends of *BTG1 *[132], *FGFRL1 *[133], and PCOTH [134] and that may be associated with non-cycling cells. Overall, there was more support at the transcriptome level for proliferation than not, which was consistent with increased proliferation observed in the LNCaP Hollow Fiber model [21].

Gene expression trends of *GLO1 *[135], *S100A10 *[136], *TRPM8 *[137], and *PI3KCD *[138] suggest cell survival pathways are active following androgen-deprivation and/or in CRPC, while gene expression trends of *CAMK2N1 *[139], *CCT2 *[140], *MDK *[141,142], *TMEM66 *[143], and *YWHAQ *[136] may oppose such suggestion. Taken together, these data neither agree nor disagree with the activation of survival pathways in CRPC. In contrast to earlier reports in which *MDK *gene and protein expression was determined to be higher in late stage cancer [63,142], we observed a drop in the levels of *MDK *mRNA in CRPC versus RAD. *MDK *expression is negatively regulated by androgen [65]. Therefore, the decreased levels of *MDK *mRNA in CRPC may suggest that the AR is reactivated in CRPC.

### Other

The significance of the gene expression trends of *AMD1, BNIP3, GRB10, MARCKSL1*, *NGRAP1, ODC1, PPP2CB, PPP2R1A, SLC25A4, SLC25A6*, and *WDR45L *that function in cell growth or cell death/survival were not straightforward. For example, *BNIP3 *and *WDR45L*, both relatively highly expressed in CRPC versus RAD, may be associated with autophagy. BNIP3 promotes autophagy in response to hypoxia [144], and the WDR45L-related protein, WIPI-49, co-localizes with the autophagic marker LC3 following amino acid depletion in autophagosomes [145]. It is not known if BNIP3 or putative WDR45L-associated autophagy results in cell survival or death. Levels of expression of *NGFRAP1 *were increased in CRPC versus RAD. The protein product of *NGFRAP1 *interacts with p75 (NTR). Together they process caspase 2 and caspase 3 to active forms, and promote apoptosis in 293T cells [146]. NGFRAP1 requires p75 (NTR) to induce apoptosis. However, LNCaP cells do not express p75 (NTR), and so it is not clear if apoptosis would occur in this cell line [147].

Overall, genes involved in cell growth and cell death pathways were altered in CRPC. Increased tumor burden may develop from a small tip in the balance when cell growth outweighs cell death. Unfortunately, the contributing weight of each gene is not known, making predictions difficult based on gene expression alone of whether proliferation and survival were represented more than cell death in this model of CRPC. It should be noted that LNCaP cells are androgen-sensitive and do not undergo apoptosis in the absence of androgens. The proliferation of these cells tends to decrease in androgen-deprived conditions, but eventually with progression begins to grow again mimicking clinical CRPC.

## Conclusion

Here, we describe the LNCaP atlas, a compilation of LongSAGE libraries that catalogue the transcriptome of human prostate cancer cells as they progress to CRPC *in vivo*. Using the LNCaP atlas, we identified differential expression of 96 genes that were associated with castration-recurrence *in vivo*. These changes in gene expression were consistent with the suggested model for a role of the AR, steroid synthesis and metabolism, neuroendocrine cells, and increased proliferation in CRPC.

## Abbreviations

*ACPP*: prostate acid phosphatise; ACTH: adrenocorticotropic hormone; AR: androgen receptor; AREs: androgen response elements; AS: androgen-sensitive; *BAX*: BCL2-associated X protein; *BCL-2*: B-cell CLL/lymphoma 2; *BCL2L1*: BCL2-like 1; CAK: cyclin-dependent activating kinase; *CCND1*: cyclin D1; CCNH: Cyclin H; *CDKN1A*: cyclin-dependent kinase inhibitor 1A; *CDKN1B*: cyclin-dependent kinase inhibitor 1B; *CHG*: chromogranin; CR: castration-recurrent; CRPC: castration-recurrent prostate cancer; CUEDC2: CUE-domain-containing-2; DHCR24: 24-dehydrocholesterol reductase; DHRS7: dehydrogenase/reductase SDR-family member 7; EASE: Expression Analysis Systematic Explorer; ELOVL5: elongation of long chain fatty acids family member 5; ENO2: neuronal enolase 2; FLNA: filamin A; GO: Gene Ontology; HES6: hairy and enhancer of split 6; HGNC: HUGO Gene Nomenclature Committee; HMGB2: high mobility group box 2; *HMGCS1*: 3-hydroxy-3-methylglutaryl-Coenzyme A synthase 1; HPA: hypothalamus-pituitary-adrenal; *HSD17B3*: hydroxysteroid (17-beta) dehydrogenase 3; HSD17B4: hydroxysteroid (17-beta) dehydrogenase 4; *HSD17B5*: hydroxysteroid (17-beta) dehydrogenase 5; IL6: interleukin 6; KEGG: Kyoto Encyclopedia of Genes and Genomes; *KLK3*: kallikrein 3; KPNB1: karyopherin/importin beta 1; LHRH: Leutinizing hormone releasing hormone; LongSAGE: long serial analysis of gene expression; MAOA: monoamine oxidase A; *NCOA*: nuclear receptor coactivator; *NKX3-1*: NK3 homeobox 1; *NTS*: neurotensin; OPRK1: opioid receptor kappa 1; PKA: protein kinase A; PSA: prostate-specific antigen also known as KLK3; PSMA7: proteasome macropain subunit alpha type 7; PTHrP: parathyroid hormone-related protein; qRT-PCR: quantitative real time-polymerase chain reaction; RAD: responsive to androgen-deprivation; RAMP1: receptor (calicitonin) activity modifying protein 1; *RB1*: retinoblastoma 1; S100A10: S100 calcium binding protein A10; *SQLE*: squalene epoxidase; TRPM8: transient receptor potential cation channel subfamily M member 8.

## Competing interests

The authors declare that they have no competing interests.

## Authors' contributions

TLR and MDS conceived, designed, conducted, and analyzed all experiments described in this manuscript. TLR and MDS wrote the manuscript. GW performed the principle component analysis. MAM was responsible for SAGE library construction and sequencing. OM (tag clustering) and AD (library clustering) aided in bioinformatic analysis. All authors read and approved the final manuscript.

## Author's information

M.D.S. and M.A.M. are Terry Fox Young Investigators. M.A.M. is a Senior Scholar of the Michael Smith Foundation for Health Research.

## Pre-publication history

The pre-publication history for this paper can be accessed here:

http://www.biomedcentral.com/1755-8794/3/43/prepub

## Supplementary Material

Additional file 1 **Supplementary Figures**. **Figure S1**: qRT-PCR analysis of *KLK3 *gene expression during hormonal progression of prostate cancer to castration-recurrence. RNA samples were retrieved from the *in vivo *LNCaP Hollow Fiber model at different stages of cancer progression that were: AS, androgen-sensitive, day zero (just prior to surgical castration and 7 days post-fiber implantation); RAD, responsive to androgen-deprivation, 10 days post-surgical castration; and CR, castration-recurrent, 72 days post-surgical castration. MNE, mean normalized expression, calculated by normalization to glyceraldehyde-3-phosphate (*GAPDH*). Error bars represent ± standard deviation of technical triplicates. Each mouse represents one biological replicate. **Figure S2**: Ten K-means clusters are optimal to describe the expression trends present during progression to castration-recurrence. K-means clustering was conducted over a range of K (number of clusters) from K = 2 to K = 20 and the within-cluster dispersion was computed for each clustering run and plotted against K. The within-cluster dispersion declined with the addition of clusters and this decline was most pronounced at K = 10. The graph of within cluster dispersion versus K shown here is for mouse 13N, but the results were similar for mice 15N and 13R. **Figure S3**: Trend legend for Table 4. Gene expression trends of LongSAGE tags that consistently and significantly altered expression in CR prostate cancer are represented graphically with trends labeled A-P. * Statistics according to the Audic and Claverie test statistic (p ≤ 0.05). Click here for file
