# Supplementary figures and images for: LNCaP Atlas: Gene expression associated with in vivo progression to castration-recurrent prostate cancer
Source: BMC Med Genomics. 2010 Sep 24;3:43. doi: 10.1186/1755-8794-3-43 (PMC2956710; doi:10.1186/1755-8794-3-43)

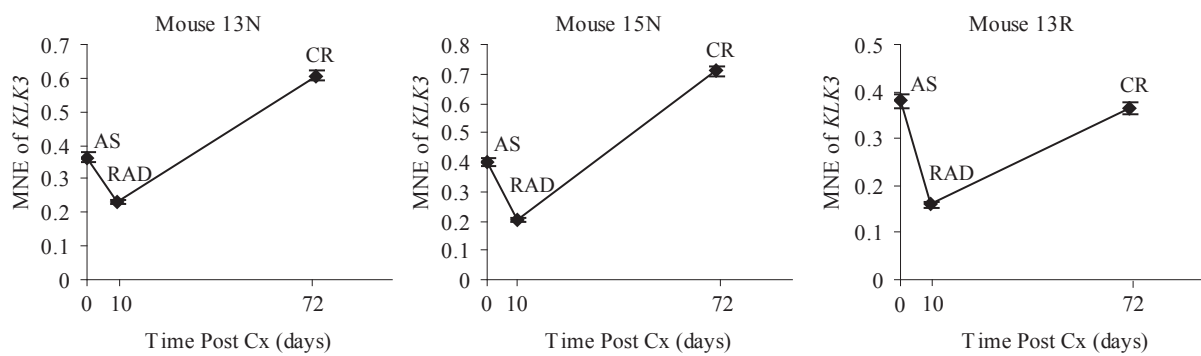

Figure S1

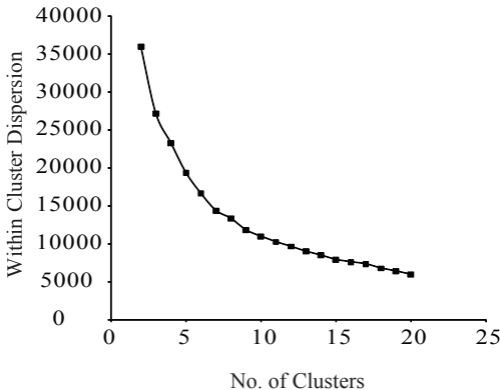

Figure S2

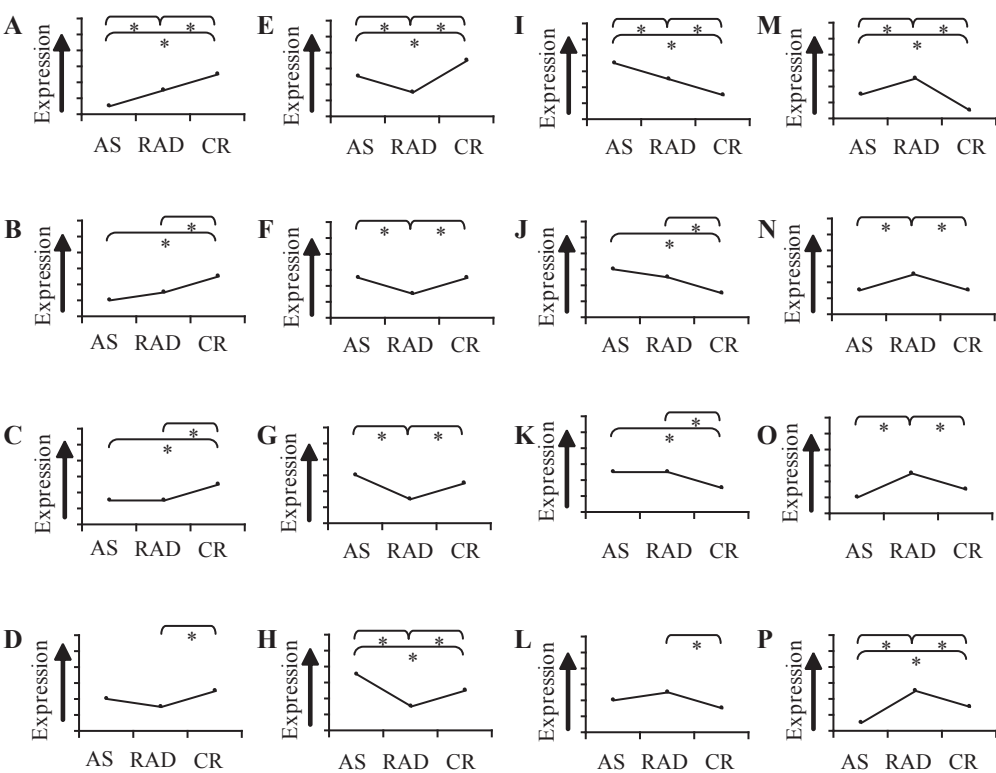

Figure S3

Supplement: Additional file 1 — Supplementary Figures. Figure S1: qRT-PCR analysis of KLK3 gene expression during hormonal progression of prostate cancer to castration-recurrence. RNA samples were retrieved from the in vivo LNCaP Hollow Fiber model at different stages of cancer progression that were: AS, androgen-sensitive, day zero (just prior to surgical castration and 7 days post-fiber implantation); RAD, responsive to androgen-deprivation, 10 days post-surgical castration; and CR, castration-recurrent, 72 days post-surgical castration. MNE, mean normalized expression, calculated by normalization to glyceraldehyde-3-phosphate (GAPDH). Error bars represent ± standard deviation of technical triplicates. Each mouse represents one biological replicate. Figure S2: Ten K-means clusters are optimal to describe the expression trends present during progression to castration-recurrence. K-means clustering was conducted over a range of K (number of clusters) from K = 2 to K = 20 and the within-cluster dispersion was computed for each clustering run and plotted against K. The within-cluster dispersion declined with the addition of clusters and this decline was most pronounced at K = 10. The graph of within cluster dispersion versus K shown here is for mouse 13N, but the results were similar for mice 15N and 13R. Figure S3: Trend legend for Table 4. Gene expression trends of LongSAGE tags that consistently and significantly altered expression in CR prostate cancer are represented graphically with trends labeled A-P. * Statistics according to the Audic and Claverie test statistic (p ≤ 0.05). [file 1755-8794-3-43-S1.PDF]
